# The Halo Effect: Suppression of Pink Bollworm on Non-Bt Cotton by Bt Cotton in China

**DOI:** 10.1371/journal.pone.0042004

**Published:** 2012-07-27

**Authors:** Peng Wan, Yunxin Huang, Bruce E. Tabashnik, Minsong Huang, Kongming Wu

**Affiliations:** 1 State Key Laboratory for Biology of Plant Diseases and Insect Pests, Institute of Plant Protection, Chinese Academy of Agricultural Sciences, Beijing, People’s Republic of China; 2 Institute of Plant Protection and Soil Science, Hubei Academy of Agricultural Sciences, Wuhan, People’s Republic of China; 3 School of Resource and Environmental Science, Hubei University, Wuhan, Hubei, People’s Republic of China; 4 Department of Entomology, University of Arizona, Tucson, Arizona, United States of America; Universidad Nacional Autonoma de Mexico, Instituto de Biotecnologia, Mexico

## Abstract

In some previously reported cases, transgenic crops producing insecticidal proteins from *Bacillus thuringiensis* (Bt) have suppressed insect pests not only in fields planted with such crops, but also regionally on host plants that do not produce Bt toxins. Here we used 16 years of field data to determine if Bt cotton caused this “halo effect” against pink bollworm (*Pectinophora gossypiella*) in six provinces of the Yangtze River Valley of China. In this region, the percentage of cotton hectares planted with Bt cotton increased from 9% in 2000 to 94% in 2009 and 2010. We found that Bt cotton significantly decreased the population density of pink bollworm on non-Bt cotton, with net decreases of 91% for eggs and 95% for larvae on non-Bt cotton after 11 years of Bt cotton use. Insecticide sprays targeting pink bollworm and cotton bollworm (*Helicoverpa armigera*) decreased by 69%. Previously reported evidence of the early stages of evolution of pink bollworm resistance to Bt cotton in China has raised concerns that if unchecked, such resistance could eventually diminish or eliminate the benefits of Bt cotton. The results reported here suggest that it might be possible to find a percentage of Bt cotton lower than the current level that causes sufficient regional pest suppression and reduces the risk of resistance.

## Introduction

Transgenic corn and cotton producing insecticidal proteins from *Bacillus thuringiensis* (Bt) were planted on more than 66 million hectares worldwide in 2011 to control insect pests and reduce reliance on insecticide sprays [1], [2].The primary threat to the continued success of these Bt crops is evolution of resistance by pests [3],[4]. Field-evolved resistance causing reduced efficacy of Bt crops has been documented for some populations of several major target pests [5]–[11]. The main approach for delaying pest resistance to Bt crops is planting refuges of non-Bt host plants near Bt crops to promote survival of susceptible pests [6], [7].

One of the potential drawbacks of the refuge strategy is increased pest damage to non-Bt crop plants in refuges [11]. In some cases, however, Bt crops suppress pest populations not only in Bt crop fields, but also in nearby non-Bt crop fields [12]–[19]. This “halo effect” was predicted on theoretical grounds, because females emerging from non-Bt crops lay some of their eggs on nearby Bt crops, and the larvae hatching from such eggs suffer high mortality on the Bt crops [12]–[14]. If Bt plants account for a substantial percentage of the available host plants, regional pest populations can be greatly reduced, resulting in less damage to non-Bt plants [12]. By reducing damage to non-Bt plants, the halo effect can reduce the need for insecticide sprays on non-Bt crops and encourage compliance with the refuge strategy, thereby increasing the benefits and sustainability of Bt crops [12], [18].

The halo effect was first documented for pink bollworm, *Pectinophora gossypiella* [14], a global pest that has many potential host plants, but feeds almost exclusively on cotton in the United States and China [20], [21]. In the United States, planting of non-Bt cotton refuges was the primary strategy for delaying pink bollworm resistance to Bt cotton from 1996 to 2005 [11]. Results from modeling suggested that regional suppression of pink bollworm in non-Bt cotton would occur when the percentage of cotton planted to Bt cotton exceeded a threshold value [14]. Analysis of 10 years of field data encompassing five years before and after adoption of transgenic cotton producing Bt toxin Cry1Ac in the state of Arizona in the southwestern United States supported this idea [14]. In particular, the Arizona field data suggested that regional suppression of pink bollworm occurred when the percentage of cotton planted to Bt cotton exceeded a threshold of approximately 65% [14].

**Figure 1:**
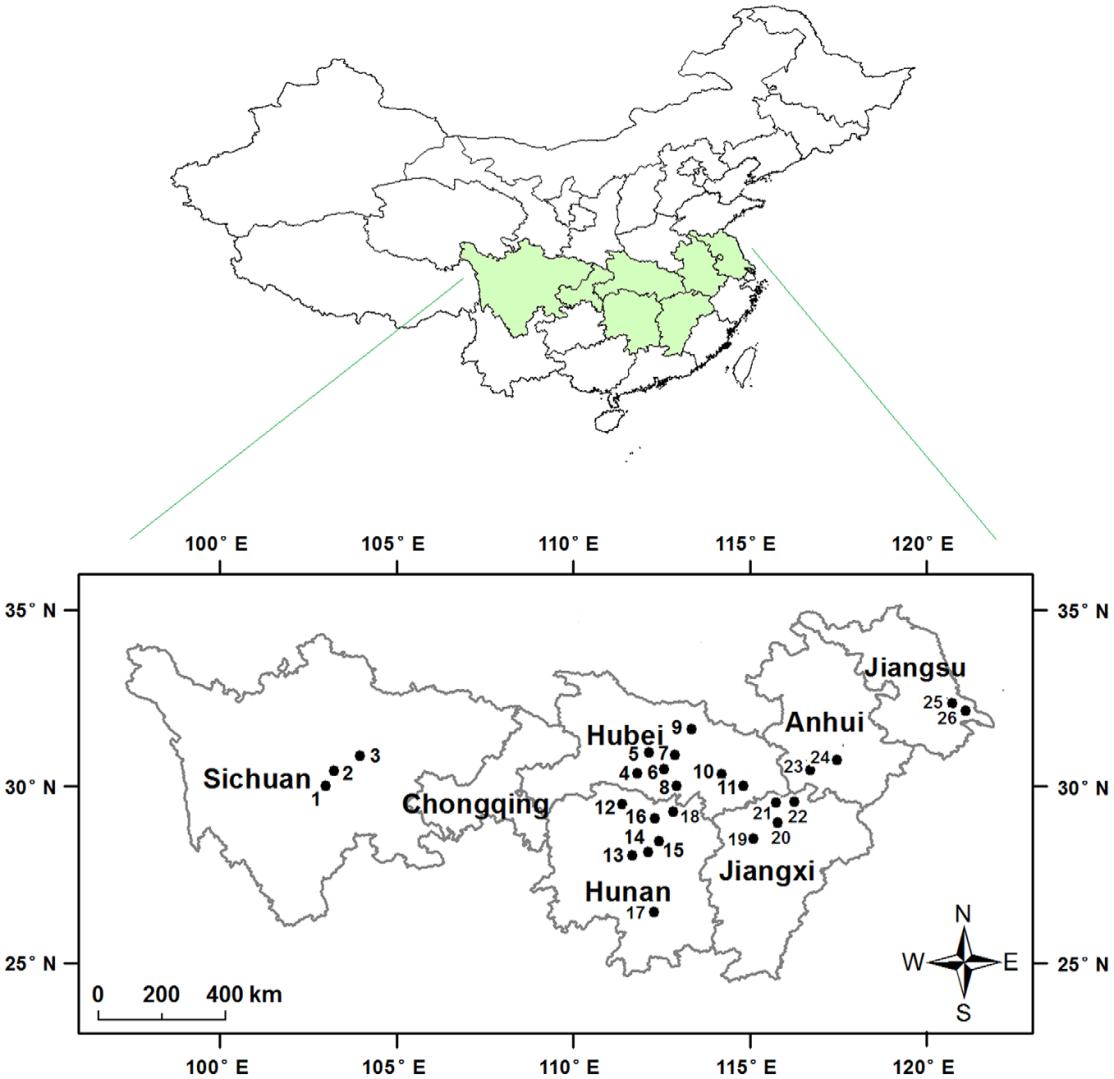
Sites for pink bollworm monitoring in China’s Yangtze River Valley.

**Figure 2:**
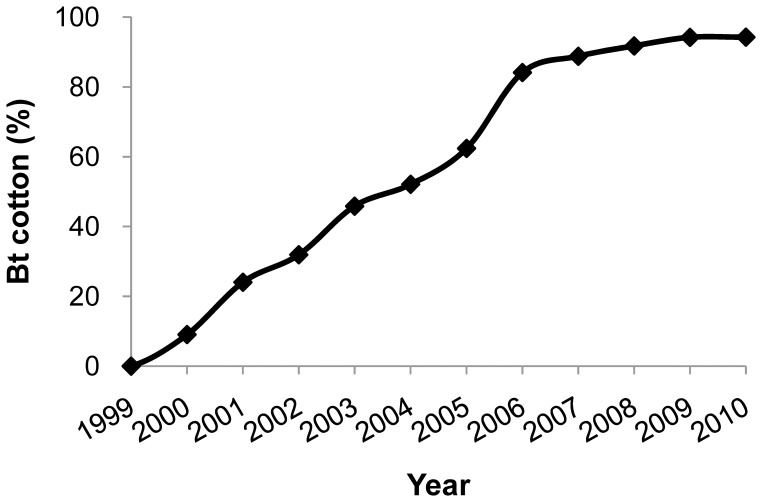
Percentage of cotton hectares planted with Bt cotton in the Yangtze River Valley, 1999 to 2010.

Here we tested the hypothesis that Bt cotton suppressed pink bollworm populations on non-Bt cotton in the Yangtze River Valley, a major cotton growing-region of China [21]. We analyzed pink bollworm population density in six provinces of the Yangtze River Valley (Fig. 1) during 16 years, including five years before Bt cotton was adopted (1995–1999) and 11 years after Bt cotton was adopted (2000–2010). In these six provinces, the percentage of cotton hectares planted with Bt cotton increased from 9% in 2000, to 62% in 2005, 84% in 2006, and 94% in 2009 and 2010 (Fig. 2). We found that the population density of pink bollworm on non-Bt cotton was 91 to 95% lower in 2010, after 11 years of Bt cotton, compared with the mean population density during the five years before Bt cotton. Consistent with results from Arizona, the annual per capita growth rate (*r*) was lower when the percentage of cotton planted to Bt cotton exceeded 65%. In addition, in 2010 compared with the eight years before Bt cotton adoption (1992–1999), insecticide sprays targeting bollworms on cotton decreased by 69%.

## Results

### Pink Bollworm Population Density and Growth Rate on Non-Bt Cotton

**Figure 3:**
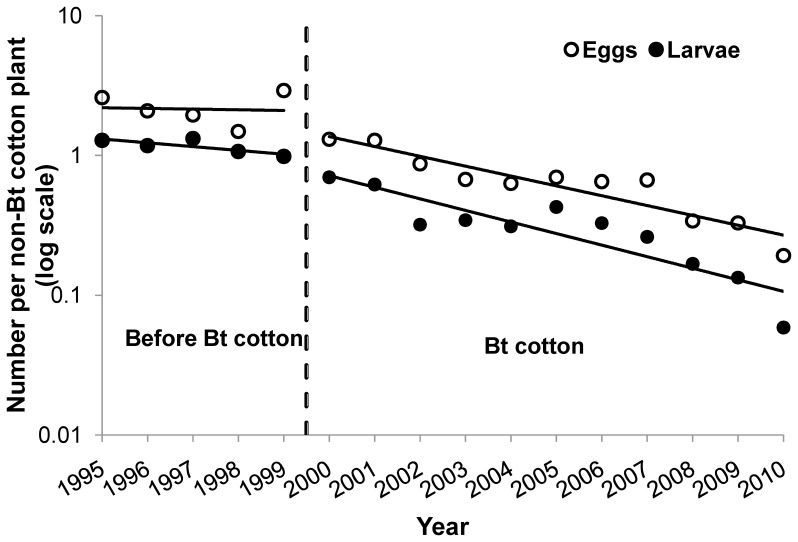
Pink bollworm abundance on non-Bt cotton before and after adoption of Bt cotton. Before Bt cotton (1995–1999), annual average abundance did not change significantly for eggs (slope = −0.0048, df = 3, *R* ^2^ = 0.004, *P*  = 0.92) and larvae (slope = −0.027, df = 3, *R^2^* = 0.64, *P*  = 0.10). With Bt cotton (2000–2010), annual average abundance declined significantly for both eggs (slope = −0.070, df = 9, *R* ^2^ = 0.86, *P*<0.0001) and larvae (slope = −0.083, df = 9, *R* ^2^ = 0.80, *P*  = 0.0002).

The abundance of both pink bollworm eggs and larvae on non-Bt cotton plants declined significantly from the first year Bt cotton was planted in the Yangtze River Valley (2000) to 2010 (Fig.3). In contrast, during the five years before Bt cotton was adopted in the Yangtze River Valley (1995–1999), the abundance of pink bollworm eggs and larvae declined somewhat, but this trend was not statistically significant (Fig. 3). Pink bollworm abundance on non-Bt cotton decreased from a mean of 2.2 eggs and 1.2 larvae per plant during 1995 to 1999 to 0.19 eggs and 0.059 larvae per plant in 2010 (Fig. 3). Compared with the mean population density for the five years before Bt cotton (1995–1999), the net decline in population density on non-Bt cotton observed in 2010, after 11 years of Bt cotton, was 91% for eggs and 95% for larvae.

From 1995 to 2010, the density of eggs and larvae on non-Bt cotton was negatively associated with the percentage of cotton planted with Bt cotton (P<0.0001 for eggs and larvae, Tables 1 and 2). By contrast, pink bollworm population density was not significantly associated with temperature or rainfall (P>0.05 for eggs and larvae, Tables 1 and 2).

**Table 1:** Regional average temperature (°C) and rainfall (*mm*) during June to October in the Yangtze River Valley from 1995 to 2010.

Year	1995	1996	1997	1998	1999	2000	2001	2002
**Temp**	25	25	24	24	25	24	25	24
**Rain**	75	72	65	64	95	65	70	66
**Year**	**2003**	**2004**	**2005**	**2006**	**2007**	**2008**	**2009**	**2010**
**Temp**	25	24	25	25	25	25	25	25
**Rain**	87	66	80	61	71	87	78	100

**Table 2:** Stepwise regression testing association of Bt cotton (%), temperature, and rainfall with population density of pink bollworm.

		Eggs				Larvae			
**Y**	**X**	**Slope**	**df**	**R^2^**	**P**	**Slope**	**df**	**R^2^**	**P**
**ln(Density)**	**Temp**	−0.41	14	0.07	0.34	−0.61	14	0.11	0.21
	**Rain**	−0.022	14	0.11	0.21	−0.34	14	0.2	0.085
	**Bt%**	−0.018	14	0.85	<0.0001	−0.021	14	0.83	<0.0001

We used data on the abundance of eggs and larvae to test the hypothesis that the annual per capita growth rate (*r*) for pink bollworm in non-Bt cotton decreased when the percentage of Bt cotton exceeded a threshold of 65% (Methods). The percentage of Bt cotton was <65% for 1995 to 2005 (mean  = 20%, range  = 0 to 62%) and >65% for 2006 to 2010 (mean  = 91%, range  = 84 to 94%) (Fig. 2). Two-way analysis of variance showed that *r* on non-Bt cotton was significantly lower with Bt cotton >65% than with Bt cotton <65% (F  = 2.9, df  = 1, 26, one-tailed P  = 0.05), but life stage (egg versus larva) and the interaction between life stage and Bt cotton (%) did not significantly affect *r* (P>0.5 for each). Whereas mean *r* with Bt cotton <65% did not differ significantly from zero (−0.12, SE  = 0.07), the mean value of *r* with Bt cotton>65% (−0.33, SE  = 0.09) indicates a 28% decrease in population density per year.

### Insecticide Sprays

**Figure 4:**
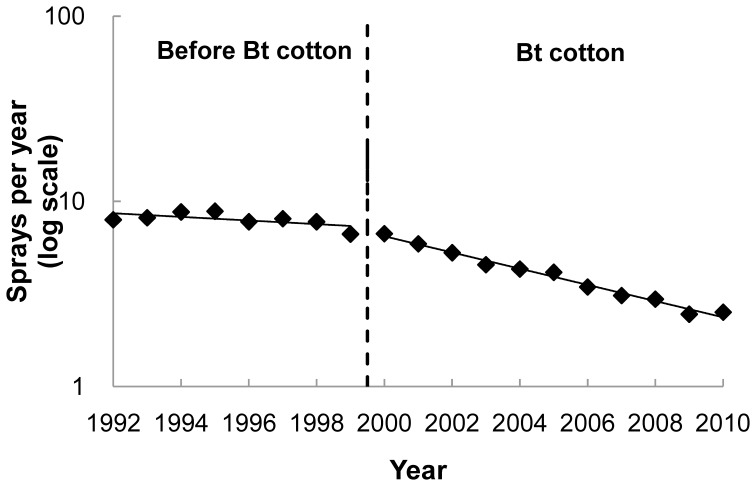
Insecticide sprays targeting bollworms on cotton per ha per year before and after adoption of Bt cotton. Before Bt cotton (1992–1999), sprays per year did not change significantly over time (slope = −0.010, df = 6, *R* ^2^ = 0.39, *P*  = 0.10). With Bt cotton (2000–2010), sprays per year declined significantly over time (slope  =  −0.044, df = 9, *R* ^2^ = 0.99, *P*<0.0001).

Before Bt cotton adoption, insecticide sprays targeting bollworms (pink bollworm and cotton bollworm, *Helicoverpa armigera*) on cotton did not change significantly from 1992 to 1999 (Fig. 4). In contrast, after Bt cotton adoption, sprays targeting bollworms declined significantly from 2000 to 2010 (Fig. 4). The mean number of sprays targeting bollworms per ha cotton per year dropped from 8.0 before Bt cotton (1992–1999) to 2.5 in 2010 (Fig. 4), which is a 69% reduction.

## Discussion

Consistent with previous results from Arizona [14], we found evidence for a halo effect of Bt cotton on pink bollworm in the Yangtze River Valley of China. Compared with the five years before Bt cotton was grown in the Yangtze River Valley (1995–1999), the population density of pink bollworm on non-Bt cotton decreased significantly during 11 years of Bt cotton use, with a net reduction of 91% for eggs and 95% for larvae (Fig. 3). Moreover, the annual per capita growth rate (*r*) was significantly lower when Bt cotton exceeded 65% of cotton planted, which is consistent with the data from Arizona [14]. Because the Bt cotton percentage in the Yangtze River Valley increased from 62% in 2005 to 84% in 2006, the results here imply that the threshold for lower *r* is somewhere between these two values.

Modeling results suggested that the threshold percentage of Bt cotton required for regional population declines becomes lower as movement of females from non-Bt cotton to Bt cotton increases and the net rate of reproduction decreases [14]. Movement of pink bollworm females from non-Bt cotton fields to Bt cotton fields is probably greater in the Yangtze River Valley than in Arizona, because cotton fields are typically <1 ha in China [21] and 15 ha in Arizona [22]. If so, this difference would favor a lower threshold for the Bt cotton percentage needed to cause population decreases in China relative to Arizona. On the other hand, the efficacy of Bt cotton against pink bollworm is lower in China than in Arizona [23], [24]. This difference, which apparently reflects lower efficacy of the GK varieties used in China relative to the Monsanto varieties used in Arizona [14], would favor a higher net rate of reproduction and a higher threshold percentage of Bt cotton needed to cause regional population declines in China relative to Arizona. The similar threshold percentage of Bt cotton causing regional population declines in Arizona and China may reflect a balance between the opposing effects of greater movement of females from non-Bt cotton to Bt cotton in China and increased survival on Bt cotton in China.

Also consistent with results from Arizona [23], [25], adoption of Bt cotton was associated with decreases in the number of insecticide sprays targeting bollworms in the Yangtze River Valley (Fig. 4). Compared with the mean during the eight years before adoption of Bt cotton in the Yangtze River Valley, the net decline in insecticide sprays targeting bollworms on cotton was 69%. This decrease in insecticide sprays targeting bollworms favored increased survival of pink bollworm, which implies that if insecticide use had not decreased, the halo effect would have caused greater suppression of pink bollworm in non-Bt cotton.

The benefits of Bt cotton in China reported here for suppressing pink bollworm on non-Bt cotton parallel previously reported benefits of Bt cotton in China for suppressing cotton bollworm on non-Bt host plants [17]. However, early evidence of resistance to the Cry1Ac toxin produced by Bt cotton grown in China has been reported for both pests [26], [27]. Options for reducing the potential for negative consequences of this resistance in China include switching to a different toxin in Bt toxin, using cotton that produces two or more toxins (preferably both distinct from Cry1Ac), using integrated pest management with tactics other than Bt cotton, and increasing the percentage of non-Bt cotton [26]–[28]. The results here suggest that it may be possible to find an optimal percentage of Bt cotton lower than the current 94% in the Yangtze River Valley that both sufficiently suppresses pink bollworm regionally and substantially reduces selection for resistance.

## Materials and Methods

### Cotton Varieties and Percentage of Cotton Planted with Bt Cotton

Bt cotton varieties planted in China are from Monsanto and the GK series developed in China (e.g., GK19 and GK12). The Monsanto varieties produce Cry1Ac and the GK varieties make a chimeric Bt toxin similar to Cry1Ac with amino acids 446–608 encoded by the *cry1Ac* gene and amino acids 1–445 encoded by the closely related *cry1Ab* gene [29]. The percentage of China's Bt cotton accounted for by GK varieties was 5% in 1998, 50% in 2003, 70% in 2005 and 93% in 2009 [30]. Each of the six cotton-growing provinces of the Yangtze River Valley has its own set of many provincial conventional cotton varieties. For example, Hubei has more than 10 conventional varieties.

For the six cotton-growing provinces of the Yangtze River Valley, we obtained the hectares planted to Bt cotton from the Chinese Agricultural Ministry (2000–2010) and the total hectares of cotton planted from the China Agriculture Yearbook. We calculated the percentage of Bt cotton for each year as the hectares of Bt cotton divided by the total hectares of cotton times 100%.

### Abundance of Pink Bollworm Eggs and Larvae

During 1995–2010, the abundance of pink bollworm eggs and larvae on cotton was monitored in six provinces of the Yangtze River Valley as part of routine national monitoring (Fig. 1). No permits were required because all collections were made in China under the auspices of the National Agro-Technical Extension and Service Center. In this region, pink bollworm has three generations per year, typically from July to October [24]. Usually, the first generation appears in July when cotton plants are flowering, the second generation occurs from early August to middle September when cotton bolls appear, and the third generation occurs in late September through October.

To measure the abundance of pink bollworm eggs and larvae, cotton plants were sampled from 8 to 21 sites across the region each year. The sites were not always the same each year; a total of 26 sites were sampled over 16 years (Fig. 1). The sites sampled for eggs were not always the same as those sampled for larvae. To measure the abundance of eggs, 100 randomly selected cotton plants were sampled from each of 10 to 20 cotton fields per site. For each sampled plant, a thorough visual whole-plant survey was conducted to count the pink bollworm eggs. The type of cotton plant (Bt or non-Bt) was not recorded. However, because pink bollworm females do not distinguish between Bt and non-Bt cotton for oviposition [31], we used the abundance of eggs on all cotton plants (Bt and non-Bt) to estimate the abundance of eggs on non-Bt cotton plants.

To measure the abundance of pink bollworm larvae in the first and second generations, 100 randomly selected non-Bt cotton plants were sampled from each of three cotton fields per site. On each plant, flowers were examined for larvae and bolls were examined for pink bollworm entry holes every 4–5 days. For the first and second generations, the number of larvae per plant was calculated as the number of larvae plus entry holes per plant. Third generation larvae were collected from harvested raw cotton [26]. In China, farmers pick cotton by hand and cotton companies buy the harvested raw cotton from farmers near their fields. When raw cotton accumulates at purchasing sites, the high temperature inside cotton bolls causes pink bollworm larvae to exit from the bolls. Therefore, it is much easier to collect larvae from raw cotton at purchasing sites than directly from cotton fields [26]. Data for the third generation were recorded as the number of larvae per kg of raw cotton. We converted this to the number of larvae per plant as follows: larvae per plant  =  (larvae per kg cotton ×kg cotton per ha)/plants per ha.

### Temperature and Rainfall

We obtained temperature and rainfall data for 1995–2010 from 27 weather stations across the Yangtze River Valley from the Chinese Meteorological Data Sharing Service System (http://cdc.cma.gov.cn/). For the 25 of 26 sampling sites with a weather station, we used the data from the weather station at the site. For the one sampling site without a weather station, we used the mean from the two nearest stations (both within 30 km of the site). We calculated mean temperature and rainfall for June to October to evaluate the impact of these factors on pink bollworm abundance (Table 1).

### Insecticide Sprays

The number of insecticide sprays targeting bollworms (pink bollworm and cotton bollworm) was recorded annually from 25–29 counties across the six provinces of the Yangtze River Valley during 1992 to 2010. Each county has one or more stations where pest incidence was monitored throughout the cotton season. When the abundance of either pink bollworm or cotton bollworm exceeded the threshold for that species [32], farmers were advised to spray their cotton fields for bollworms.

### Regional Average of Population Density

For eggs and larvae separately, we calculated the regional population density for the Yangtze River Valley for each year as follows: For each generation in each year, we calculated the provincial mean population density for all sites in the province. We calculated the annual population density for each province as the mean population density for the province for the three yearly generations. We calculated the annual regional population density for the Yangtze River Valley as the mean of the annual provincial population densities for that year.

### Annual Per Capita Growth Rate (*r*)

We calculated *r* for pink bollworm on non-Bt cotton as ln [*N_t_/N_t-1_*] [33], where *N_t_* and *N_t-1_* are the population density in year *t* and the previous year *t−1*, respectively. With 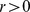,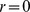 and 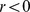, the population increases, remains unchanged, and decreases, respectively. We calculated *r* separately for eggs and larvae. We used two-way analysis of variance to test for the effects on *r* of percentage of Bt cotton (<65% vs. >65%), life stage (egg vs. larva), and the interaction of these two factors.

### Stepwise Regression

We used stepwise regression to test for significant effects on pink bollworm population density of Bt cotton (%), insecticide sprays targeting bollworms, temperature, and rainfall (*stepwise* in MATLAB 2010a). This approach determines the best regression model by stepwise addition of the most significant explanatory variables and removal of non-significant explanatory variables [34].
